# Arteriovenous malformation of the maxillary sinus: a rare clinical entity^☆^

**DOI:** 10.1016/j.bjorl.2016.08.003

**Published:** 2016-08-23

**Authors:** Dongwon Kim, Kyung-Un Choi, Hak-Jin Kim, Kyu-Sup Cho

**Affiliations:** a Pusan National University Hospital, Pusan National University School of Medicine, Department of Otorhinolaryngology and Biomedical Research Institute, Busan, Republic of Korea; b Pusan National University Hospital, Pusan National University School of Medicine, Department of Pathology, Busan, Republic of Korea; c Pusan National University Hospital, Pusan National University School of Medicine, Department of Radiology, Busan, Republic of Korea

## Introduction

Arteriovenous malformations are a structural vascular abnormality caused by the absence of normal capillary beds, which lead to the development of abnormal blood channels that connect the arterial circulation to the venous circulation.^1^ Although vascular tumors in the head and neck are relatively common, particularly in the jaw, arteriovenous malformations of the nasal cavity and paranasal sinus are extremely rare.^2^ These lesions are usually benign, but in some cases they can be life-threatening because of their potential for intractable bleeding.^1^ Furthermore, there is no consensus on the most effective surgical technique. Herein we describe a rare case of arteriovenous malformation of the maxillary sinus that presented as recurrent epistaxis after nasal polypectomy, which was completely removed via Caldwell-Luc approach without endovascular embolization. This study was approved by the institutional review board of Pusan National University Hospital.

## Case report

**Figure 1:**
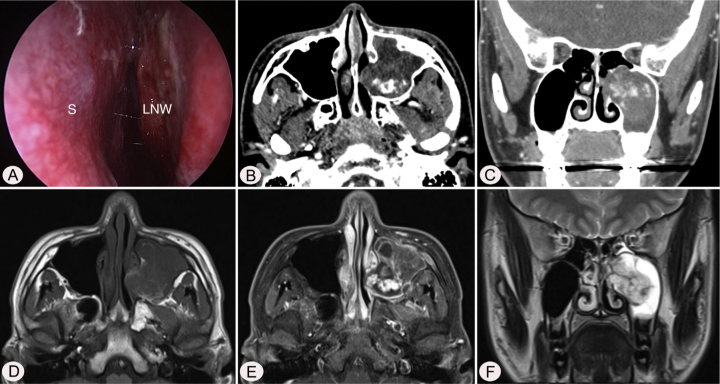
Preoperative endoscopic and radiologic findings. Nasal endoscopy (A) shows bulging of left Lateral Nasal Wall (LNW). Axial (B) and coronal (B) computed tomographic images shows heterogenous enhancing mass in left maxillary sinus without adjacent bony involvement. The mass shows mixed high signal intensity on T1 axial (D), avid enhancement on postcontrast T1 axial (E), and heterogenous high signal intensity on T2 coronal (F) magnetic resonance images. S, nasal septum.

**Figure 2:**
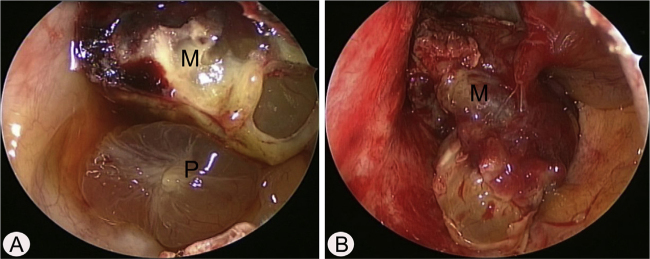
Intraoperative endoscopic findings via Caldwell-Luc approach. (A) The polypoid mucosa (P) was detected in the inferior portion of left maxillary sinus. (B) After removal of polypoid mucosa, highly vascular mass (M) was originated from the medial and partial superior wall of left maxillary sinus.

**Figure 3:**
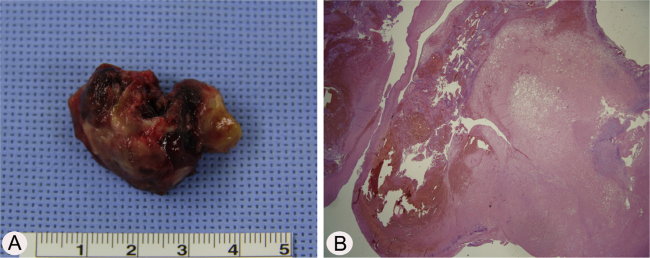
Histopathologic findings of arteriovenous malformation (A) The mass was removed via Caldwell-Luc approach and measured 3.2 cm × 2.5 cm × 1.0 cm. (B) The specimen section shows variable sized, irregular shaped vascular spaces with thrombus (H&E, ×200).

A 17-year-old male was referred to our outpatient clinic because of recurrent epistaxis after left nasal polypectomy. He had undergone nasal polypectomy due to nasal congestion at a local clinic 10 days ago. He had neither other symptoms nor any medical history. There was no history of trauma and no sign of allergy. The endoscopic examination revealed bulging of lateral nasal wall and bleeding due to left maxillary sinus mass (Fig. 1A). A Computed Tomography (CT) scan of the paranasal sinuses showed heterogenous enhancing mass in left maxillary sinus without adjacent bony involvement (Fig. 1B and C). On Magnetic Resonance Imaging (MRI), the mass expanding left maxillary sinus had mixed high signal intensity on T1-weighted images (T1WIs) and heterogenous high signal intensity on T2-weighted images (T2WIs) with an avid enhancement (Fig. 1D–F). Considering the location of tumor, surgical access was gained via Caldwell-Luc approach under general anesthesia because transnasal endoscopic sinus surgery had high risk of bleeding. The mass was originated from medial wall and partial superior wall of left maxillary sinus (Fig. 2A and B). The base of the lesion including the healthy mucosa around it was successfully removed with harmonic scalpel and microdebrider under direct visualization using a nasal endoscope and cauterized using suction cautery for prevention of recurrence (Fig. 3A). Histopathologic examination showed variable sized and irregular shaped vascular spaces with thrombus, consistent with arteriovenous malformation (Fig. 3B). Postoperative course was uneventful and he was discharged five days after surgery. Endoscopic examinations and CT performed 3 months postoperatively showed no evidence of recurrence.

## Discussion

Vascular malformations occur as a result of aberrant vessel angiogenesis during embryogenesis.^3^ Most of the head and neck vascular malformations occur in the scalp and the skin.^4^ Arteriovenous malformations are the least common of the vascular malformations and belong to a category of high-flow lesions.^5^ Although vascular malformations are congenital in nature, they may not be seen at birth. These lesions may not be evident until additional growth or vascular engorgement is seen as a response to thrombosis, trauma, infection, or endocrine fluctuations.^3^ Unlike hemangiomas, which undergo spontaneous regression, the size of arteriovenous malformations generally increases proportionately as the child grows. The mean age at presentation is 19 years and both sexes are equally affected.^6^

The most common presenting complaint of arteriovenous malformations of the maxillofacial region is chronic intermittent bleeding although some of these tumors have no signs or symptoms.^1^ Patients may present with headaches, buzzing, pain, pressure, toothache, earache, pulsation, epistaxis, swelling, facial asymmetry, ocular pain, teeth mobility, and compressibility of teeth into their sockets.^1^, ^3^ Our patient's primary symptoms was recurrent nasal bleeding after nasal polypectomy with no other symptoms. Since our patient had no history of surgery or blunt trauma, we assumed that his arteriovenous malformation was congenital. It is important to differentiate arteriovenous malformation from hemangiomas. Histologically, arteriovenous malformation is made up of aberrant arteriovenous channels in communication with each other. The abundance of arterial (thick-walled) components with different sized shapes and adjacent thin-walled venous channel can help us to diagnose it as arteriovenous malformation.^4^

Although many arteriovenous malformations may be suspected clinically, imaging is required for confirmation and treatment planning, especially as incorrect diagnosis may lead to inadequate or inappropriate treatment resulting in life threatening hemorrhage as well as recurrence.^7^ MRI is the investigation of choice as it provides accurate information about the extent of the lesion, better contrast between the lesion and surrounding tissues, and has multiplanar capabilities. It can also help distinguish between the different types of vascular anomalies.^2^, ^8^ Contrast-enhanced CT has a role in evaluating intraosseous lesions and the bony margins of extensive lesions that are under consideration of resection.^2^ Angiography, particularly digital subtraction angiography, has a specific but limited role in the diagnosis of vascular lesions, but should not be used as a first line investigation. However, it is useful for mapping out the blood supply of the lesion and in the assessment of the characteristics of flow of arteriovenous malformations.^2^ Angiography is usually reserved for therapeutic endovascular interventions.

Complete surgical resection with or without preoperative intra-arterial embolization is necessary to prevent recurrence. Embolization is indicated for occlusion of unresectable lesions, staged occlusion as preparation for resection, or as an emergent preoperative hemostatic control. Preoperative embolization may help delineate the extent of a lesion, reduce intraoperative bleeding, and lessen the risk of recurrence. Because collateral blood supply becomes established quickly, operation should be within 24–48 h after embolization. Furthermore, no patients with arteriovenous malformations were cured by simple embolization.^5^ The aim of surgery is complete removal of the nidus, which is the fundamental abnormality as even the smallest residual nidus will expand to form recurrence.^2^ The choice of endoscopic or external approach depends on the type of malformation (vascular content), age at treatment, location, depth, and extent of the lesion.^2^ Although the effective surgical management of high-flow lesions without preoperative embolization has been described,^9^ resection after embolization has become the most accepted treatment. Surgical treatment without previous embolization is more effective for smaller, well-localized focal lesions because they are likely to have smaller feeder vessels and well-defined borders.^10^ They also have a better chance of cure, and there is less likelihood of intraoperative hemorrhage.^11^ The CT and MRI findings of the lesion in this case suggested a surgical approach, which was chosen based on the area involved. We successfully removed the tumor via Caldwell-Luc approach without preoperative embolization, because it was not large lesions without bony involvement.

Diffuse arteriovenous malformations cross and destroy tissue boundaries, and have a high rate of recurrence after surgical or embolic therapy.^12^ The recurrence rate after surgical resection was 81% and 98% after embolization.^13^ Suggested recurrence mechanisms include a proangiogenetic environment involving hypoxia, trauma, and inflammation, and recanalization of the nidus vasculature. If any nidus remnant remains, these factors will lead to a recurrence, often with complex architecture and extensive vascular recruitment.^13^ In the present case, the nidus of arteriovenous malformation in the maxillary sinus was completely removed via Caldwell-Luc approach without significant bleeding.

## Conclusion

Although arteriovenous malformation in the maxillary sinus represents a rare pathology, it is important to consider this in the differential diagnosis of patients with recurrent epistaxis from a highly vascular mass of the maxillary sinus. Complete surgical removal via Caldwell-Luc approach may be considered for this lesion if possible, with preservation of cosmesis and function when surgical intervention is indicated.

## Conflicts of interest

The authors declare no conflicts of interest.

## References

[1] Cansiz H., Yener M., Kalekoglu N., Dalkilic O. (2003). Arteriovenous malformation of the maxillary sinus and mandible: a case report. Ear Nose Throat J.

[2] Ethunandan M., Mellor T.K. (2006). Haemangiomas and vascular malformations of the maxillofacial region-a review. Br J Oral Maxillofac Surg.

[3] Kademani D., Costello B.J., Ditty D., Quinn P. (2004). An alternative approach to maxillofacial arteriovenous malformations with transosseous direct puncture embolization. Oral Surg Oral Med Oral Pathol Oral Radiol Endod.

[4] Coskun B.U., Sozen E., Basak T., Alkan S., Dadas B. (2005). Arteriovenous malformation of the nasopharynx: a case report. Int J Pediatr Otorhinolaryngol.

[5] Chen W., Wang J., Li J., Xu L. (2005). Comprehensive treatment of arteriovenous malformations in the oral and maxillofacial region. J Oral Maxillofac Surg.

[6] Kohout M.P., Hansen M., Pribaz J.J., Mulliken J.B. (1998). Arteriovenous malformations of the head and neck: natural history and management. Plast Reconstr Surg.

[7] Bittles M.A., Sidhu M.K., Sze R.W., Finn L.S., Ghioni V., Perkins J.A. (2005). Multidetector CT angiography of pediatric vascular malformations and hemangiomas: utility of 3-D reformatting in differential diagnosis. Pediatr Radiol.

[8] Konez O., Burrows P.E. (2002). Magnetic resonance of vascular anomalies. Magn Reson Imaging Clin N Am.

[9] Nair S.C., Spencer N.J., Nayak K.P., Balasubramaniam K. (2011). Surgical management of vascular lesions of the head and neck: a review of 115 cases. Int J Oral Maxillofac Surg.

[10] Goldenberg D.C., Hiraki P.Y., Caldas J.G., Puglia P., Marques T.M., Gemperli R. (2015). Surgical treatment of extracranial arteriovenous malformations after multiple embolizations: outcomes in a series of 31 patients. Plast Reconstr Surg.

[11] Uller W., Alomari A.I., Richter G.T. (2014). Arteriovenous malformations. Semin Paediatr Surg.

[12] Hoff S.R., Rastatter J.C., Richter G.T. (2015). Head and neck vascular lesions. Otolaryngol Clin North Am.

[13] Liu A.S., Mulliken J.B., Zurakowski D., Fishman S.J., Greene A.K. (2010). Extracranial arteriovenous malformations: natural progression and recurrence after treatment. Plat Reconstr Surg.

